# Prevalence of multidrug resistance in *Mycobacterium tuberculosis* isolates from HIV seropositive and seronegative patients with pulmonary tuberculosis in north India

**DOI:** 10.1186/1471-2334-13-137

**Published:** 2013-03-15

**Authors:** Sunil Sethi, Abhishek Mewara, Sunil Kumar Dhatwalia, Harpal Singh, Rakesh Yadav, Khushwinder Singh, Dheeraj Gupta, Ajay Wanchu, Meera Sharma

**Affiliations:** 1 Department of Medical Microbiology, Postgraduate Institute of Medical Education and Research, Chandigarh 160012, India; 2 Department of Pulmonary Medicine, Postgraduate Institute of Medical Education and Research, Chandigarh, India; 3 Department of Internal Medicine, Postgraduate Institute of Medical Education and Research, Chandigarh, India

**Keywords:** Mycobacterium tuberculosis, Multidrug resistant, Extensively-drug resistant, HIV, Socioeconomic status

## Abstract

**Background:**

Multidrug resistant (MDR) and extensively-drug resistant (XDR) tuberculosis (TB) are a serious threat to the national TB control programs of developing countries, and the situation is further worsened by the human immunodeficiency virus (HIV) pandemic. The literature regarding MDR/XDR-TB is, however, scanty from most parts of India. We carried out this study to assess the prevalence of MDR/XDR-TB in new and previously treated cases of pulmonary TB and in HIV seropositive and seronegative patients.

**Methods:**

Sputum and blood specimens were obtained from 2100 patients suspected of pulmonary tuberculosis and subjected to sputum microscopy and culture for TB, and HIV serology at our tertiary care centre in north India. The culture positive *Mycobacterium tuberculosis* isolates were subjected to drug susceptibility testing (DST) for first line anti-tuberculosis drugs, and the MDR isolates were further subjected to second line DST. Various parameters of the patients’ were analyzed viz. clinical presentation, radiology, previous treatment history, demographic and socioeconomic data and microbiology results.

**Results:**

Of the 2100 patients, sputum specimens of 256 were smear positive for acid-fast bacilli (AFB), 271 (12.9%) grew *Mycobacterium* spp., and *M. tuberculosis* was isolated in 219 (10.42%). Of the 219 patients infected with *M. tuberculosis*, 20.1% (44/219) were found to be seropositive for HIV. Overall, MDR-TB was observed in 17.4% (39/219) isolates. There were 121 newly diagnosed and 98 previously treated patients, of which MDR-TB was found to be associated with 9.9% (12/121) and 27.6% (27/98) cases respectively. There was significantly higher association of MDR-TB (12/44, 27.3%) with HIV seropositive patients as compared to HIV seronegative patients (27/175, 15.4%) after controlling previous treatment status, age, and sex (odd’s ratio, 2.3 [95% CI, 1.000-5.350]; p-value, 0.05). No XDR-TB was found among the MDR-TB isolates.

**Conclusion:**

The present study demonstrated a high prevalence of drug resistance amongst pulmonary TB isolates of *M. tuberculosis* from north India as compared to the WHO estimates for India in 2010, though this could possibly be attributed to the clustering of more serious or referred cases at our tertiary care centre. The prevalence of MDR-TB in HIV seropositive patients was significantly higher than seronegative individuals. The study emphasizes the need to monitor the trends of drug resistance in TB in various populations in order to timely implement appropriate interventions to curb the menace of MDR-TB.

## Background

At the 2007 World Health Assembly, WHO recognized the importance of the situation and trends of multidrug resistant (MDR) and extensively drug resistant (XDR) strains of *Mycobacterium tuberculosis* as barriers to the achievement of the WHO’s Global Plan’s objectives by 2015. Among the 8.8 million incident cases of Tuberculosis (TB), 3.6% are estimated to have MDR-TB per year globally, and among the world’s 12.0 million prevalent cases of TB, 650000 are estimated to be MDR-TB cases [1]. Also, 58 countries have reported at least one case of XDR-TB as of March 2010, and it is estimated that about 5.4% of MDR-TB cases have XDR-TB.

According to WHO, nearly 50% of the world’s burden of MDR-TB cases is in India and China [2]. The situation of TB is further threatened by the devastating effect of human immunodeficiency virus (HIV) on tuberculosis susceptibility and the rapid expansion of MDR-TB threaten to undermine the advances made by tuberculosis management programs [3]. Tuberculosis is one of the earliest opportunistic diseases to develop amongst persons infected with HIV, and HIV infection is the most powerful risk factor of progression of TB from infection to disease with 21–34 times more chances to develop the disease as compared to those who are HIV-negative. Globally, just over one in ten of the almost 9 million people who develop TB each year are HIV-positive, equivalent to 1.1 million new TB cases among people living with HIV [1].

MDR and XDR-TB are a serious threat to the national tuberculosis control programs of developing countries. The chances of patients with MDR-TB being cured are low and they require significant expenditure of health care resources. The level of drug resistance is said to provide an epidemiological indicator to assess the amount of transmission of resistant bacteria in the community, as the patients harbouring resistant bacilli remain infectious for a prolonged period and may be more likely to infect others [4]. Hence there is a need to strengthen the surveillance for drug-resistant TB to ensure continuous monitoring of the epidemiological profile of MDR and XDR-TB. In India, it becomes difficult to determine the exact magnitude of the problem of drug resistance as majority of the laboratories providing services for diagnosis of TB do not perform cultures of TB and there are only a limited number of laboratories capable of conducting quality assured first and second line drug susceptibility testing (DST).

From India, though drug resistance in TB has frequently been reported, most of the available information is localized, sketchy or incomplete, and most studies have used non-standardized methodologies to assess drug resistance [5]. A large scale population based survey in the states of Gujarat and Maharashtra has indicated multidrug resistance levels of <3% among new TB cases and 12-18% among previously treated TB patients [6]. The literature regarding MDR/XDR-TB is scanty in most of the other regions of the country. The present study was hence planned to assess the prevalence of MDR/XDR-TB in patients with clinical suspicion of pulmonary TB presenting to our tertiary care centre which caters to a large population of patients from several states of north India (Chandigarh, Punjab, Haryana, Himachal Pradesh, Uttar Pradesh, Uttaranchal, Jammu and Kashmir, and some parts of Rajasthan), with the objective to evaluate the resistance patterns in new and previously treated cases, and in HIV seropositive and seronegative individuals. Many of these patients are partially treated, and non compliance is a major factor which may contribute to development of resistance to anti-tuberculosis drugs.

## Methods

The study was prospectively carried out from October, 2006 to February, 2010 for a span of 41 months. Sputum and blood samples were obtained from 2100 patients suspected of pulmonary TB attending the chest and medical out patient’s departments (OPDs) of our tertiary care centre, the Postgraduate Institute of Medical Education and Research (PGIMER), Chandigarh, India. The patients included had clinical signs and symptoms suggestive of pulmonary TB (cough, fever, weight loss, breathlessness, chest pain, hemoptysis), with/without radiological evidences (exudation, infiltration, cavitation, calcification, pleural effusion) and a family history of pulmonary TB. All the tests were conducted at the Mycobacteriology laboratory of PGIMER which is a RNTCP (Revised National TB Control Programme) approved centre for tuberculosis culture and drug sensitivity testing accredited by Central TB Division, Ministry of Health of the Government of India. A written informed consent was taken from all the patients and the study was approved by the Institute Ethics Committee of PGIMER, Chandigarh.

The patients were categorized into seven age groups: <11, 11–20, 21–30, 31–40, 41–50, 51–60, > 60 years of age. The socio-economic status of the patients was recorded based on their occupations; the lower socio-economic class comprised of the agricultural workers, manual labourers, household workers and unemployed people, and the upper socio-economic class comprised of salaried government or private professionals, and businessmen.

A new case of TB was defined as a patient who has never had treatment for TB or who has taken anti-TB drugs for less than one month. Retreatment case of TB were of three types: (i) a patient previously treated for TB, who is started on a retreatment regimen after previous treatment has failed (treatment after failure); (ii) a patient previously treated for TB who returns to treatment having previously defaulted; and (iii) a patient who was previously declared cured or treatment completed and is diagnosed with bacteriologically-positive (sputum smear or culture) TB (relapse). A case of MDR-TB was defined as TB that is resistant to two first-line drugs: isoniazid and rifampicin [1]. XDR-TB was defined as MDR-TB plus resistance to a fluoroquinolone and at least one second-line injectable agent: amikacin, kanamycin and/or capreomycin [2].

### Specimen collection and transport

#### Sputum

Two sputum samples from each patient were collected in wide mouthed sterile capped containers and were transported to the laboratory within an hour and processed immediately. In case of delay, the samples were stored at 4°C till processing.

#### Blood

From each patient 5–7 ml of blood was collected by venipuncture. The serum was separated in the laboratory and HIV screening was performed according to NACO guidelines [7]. The serum was then stored at −20°C.

### Sputum microscopy and culture

Sputum samples were subjected to Ziehl Neelsen’s acid fast staining (ZN staining) to look for the presence of acid fast bacilli (AFB). Samples were decontaminated by N-Acetyl-L-Cysteine Sodium Hydroxide (NALC-NaOH) method and cultured on LJ media, and identified according to standard methods [8]. Inoculated cultures were incubated at 37°C for 6 weeks and examined every week to look for the presence of mycobacterial growth. Any suspected growth on LJ medium was confirmed by ZN staining for AFB, and the positive growths were sub-cultured on fresh LJ media for further identification and sensitivity.

### Biochemical identification

Identification of mycobacteria isolated after primary culture on LJ medium was done by growth rate, colony morphology, colour, niacin production, nitrate reduction, catalase production at 68°C, and growth on LJ with PNB (500 mg/ml).

### Drug susceptibility testing (DST)

DST was carried out for first line anti-tuberculosis drugs, namely rifampicin (40 μg/ml), isoniazid (1 μg/ml), ethambutol (2 μg/ml) and streptomycin (4 μg/ml) using the standard proportion method [9]. Briefly, growth from positive LJ slopes was suspended in normal saline and vortexed for 10–15 minutes. The suspension was allowed to settle for 15–20 minutes and compared with McFarland standard number one. Then, 1/100 and 1/10000 dilutions of above suspension were prepared in normal saline and used as inoculums. For each drug and for each dilution, 100 μl of the inoculum was used to inoculate two drug-free and two drug-containing LJ medium. Standard reference strain H37Rv was used as control with each batch of sensitivity. The MDR isolates were further screened for XDR-TB by performing second line DST for ofloxacin (2 μg/ml), amikacin (1 μg/ml), kanamycin (30 μg/ml), and capreomycin (40 μg/ml) in liquid culture by BACTEC MGIT 960 [10].

### Statistical analysis

For demographic and DST data, frequencies, percentages, means, medians, ranges, and confidence intervals (CI) were calculated as appropriate. The differences in groups were analysed by χ^2^ test. The association of MDR-TB with HIV was analysed by multivariable regression analysis. A two-sided p-value of less than 0.05 was considered as statistically significant. The data was analyzed using the SPSS version 15.0 software.

## Results

### Study population

Sputum and blood specimens were obtained from a total of 2100 patients clinically suspected of pulmonary tuberculosis prospectively enrolled in the study. There were 70.28% (1476) males and 29.71% (624) females. Patients of all age groups were enrolled, and majority of the patients i.e. 70.85% (1488/2100) were between 21 to 50 years of age, though there was no significant preponderance of TB in any specific age group (Table 1).

**Table 1:** **Age wise distribution of *M. tuberculosis *and MDR-TB isolates**

	**Total**			**Males**			**Females**		
**Age**	**N**	**MTB**	**MDR**	**N**	**MTB**	**MDR**	**N**	**MTB**	**MDR**
**<11**	29	1	0	18	0	0	11	1	0
**11-20**	219	24	8	137	16	4	82	8	4
**21-30**	501	52	10	338	34	6	163	18	4
**31-40**	568	71	15	403	51	11	165	20	4
**41-50**	419	39	4	307	28	3	112	11	1
**51-60**	228	23	1	170	18	1	58	5	0
**>60**	136	9	1	103	8	1	33	1	0
**Total**	**2100**	**219**	**39**	**1476**	**155**	**26**	**624**	**64**	**13**

MDR – multidrug resistant, MTB – *M. tuberculosis*, N – number.

### Microscopy and culture

Of the 2100 patients, sputum specimens from 256 (12.19%) were found positive for AFB, while the culture grew *Mycobacteria* spp. in 271 (12.9%). Culture positivity in smear positive cases was found to be 80.8% (207/256) and culture positivity in smear negative cases was 3.4% (64/1844). *M. tuberculosis* and non-tuberculous mycobacteria (NTM) grew from 10.42% (219) and 2.47% (52) cases, respectively. The 52 NTM isolates (18 from HIV positive and 34 from HIV seronegative) were not analyzed further. The 219 *M. tuberculosis* isolates were subjected to DST.

Among the 219 culture positive *M. tuberculosis* isolates there were 121 newly diagnosed cases that had never taken treatment for TB or had taken anti-TB treatment for less than one month, while 98 patients gave a history of previous anti-TB treatment more than one month, of which 39 were treatment failures, 21 relapsed after treatment, 13 defaulted during the therapy course, and 25 patients did not provide sufficient history to be categorized into any group.

### Antimicrobial resistance

The drug resistance observed to any of the first line drugs in new and previously treated patients was 26.4% (95% CI, 19.4–35.4) and 46.9% (95% CI, 37.1–56.8) to isoniazid, 9.9% (95% CI, 5.8–16.5) and 27.6% (95% CI, 18.7–36.4) to rifampicin, 14.9% (95% CI, 9.6–22.3) and 33.7% (95% CI, 24.3–43.0) to ethambutol, and 28.1% (95% CI, 20.9–36.7) and 34.7% (95% CI, 25.3–44.1) to streptomycin, respectively. The total mono-resistance in new and previously treated patients was found to be 20.7% (95% CI, 13.4–27.9) and 32.6% (95% CI, 23.4–41.9), respectively. Table 2 shows the resistance profile of the *M. tuberculosis* isolates.

**Table 2:** First line anti-tuberculosis drug resistance in new and previously treated patients

**Total number of specimens = 2100**		**Previous anti-TB treatment status**					
		**New**		**Previously treated**		**Total**	
		**N**	**% (95% CI)**	**N**	**% (95% CI)**	**N**	**% (95% CI)**
**Total DST results**		**121**	**5.8**	**98**	**4.7**	**219**	**10.4**
**I**	Any resistance to H	32	26.4 (19.4–35.4)	46	46.9 (37.1–56.8)	78	35.6 (29.3–42.0)
	Any resistance to R	12	9.9 (5.8–16.5)	27	27.6 (18.7–36.4)	39	17.8 (12.7–22.9)
	Any resistance to E	18	14.9 (9.6–22.3)	33	33.7 (24.3–43.0)	51	23.3 (17.7–28.9)
	Any resistance to S	34	28.1 (20.9–36.7)	34	34.7 (25.3–44.1)	68	31.1 (24.9–37.2)
**II**	Resistance to H only	10	8.3 (4.7–15.3)	14	14.3 (7.1–20.9)	24	10.9 (6.8–15.1)
	Resistance to R only	0	0 (0–0)	0	0 (0–0)	0	0 (0–0)
	Resistance to E only	0	0 (0–0)	7	7.1 (1.9–12.1)	7	3.1 (0.9–5.5)
	Resistance to S only	15	12.4 (8.6–21.4)	11	11.2 (4.8–17.2)	26	11.9 (7.6–16.2)
**Total mono-resistance**		**25**	**20.7 (13.4–27.9)**	**32**	**32.6 (23.4–41.9)**	**57**	**26.02 (20.2-31.9)**
**III**	H + R	1	0.8 (−0.8–2.8)	6	6.1 (1.3–10.7)	7	3.1 (0.9–5.5)
	H + R + E	3	2.5 (0.04–6.04)	7	7.1 (1.9–12.1)	10	4.5 (1.8–7.3)
	H + R + S	3	2.5 (0.04–6.04)	5	5.1 (0.7–9.3)	8	3.7 (1.2–6.2)
	H + R + E + S	5	4.1 (1.1–8.9)	9	9.2 (3.3–14.7)	14	6.4 (3.2–9.6)
**Total MDR**		**12**	**9.9 (4.6–15.2)**	**27**	**27.6 (18.7–36.4)**	**39**	**17.8 (12.7–22.9)**
**IV**	H + E	4	3.3 (0.5–7.5)	3	3.1 (−0.4–6.4)	7	3.1 (0.9–5.5)
	H + S	5	4.1 (1.1–8.9)	2	2.04 (−0.8–4.8)	7	3.1 (0.9–5.5)
	H + E + S	1	0.8 (0.8–2.8)	3	3.1 (−0.4–6.4)	4	1.8 (0.1–3.6)
	R + E	0	0 (0–0)	0	0 (0–0)	0	0 (0–0)
	R + S	0	0 (0–0)	0	0 (0–0)	0	0 (0–0)
	R + E + S	0	0 (0–0)	0	0 (0–0)	0	0 (0–0)
	E + S	5	4.1 (1.1–8.9)	4	4.1 (0.12–7.9)	9	4.1 (1.5–6.7)
**Total poly-resistance other than MDR**		**15**	**12.4 (6.8–18.3)**	**12**	**12.2 (5.8–18.7)**	**27**	**12.3 (8.0–16.7)**

CI – confidence interval, DST – drug susceptibility testing, E – ethambutol, H – isoniazid, R – rifampicin, S – streptomycin.

Overall, resistance to both isoniazid and rifampicin (MDR) was observed in 17.8% (39/219; 95% CI, 12.7–22.9) of the isolates. All the MDR strains were subjected for susceptibility to second line anti-tubercular drugs, however, no XDR strain was found in our study. Of the 39 MDR-TB isolates, the resistance to second line drugs was 15.4% (6/39) to ofloxacin, 7.7% (3/39) to kanamycin, and 2.5% (1/39) to amikacin, while no resistance to capreomycin was observed. Among the new and previously treated cases, MDR-TB was found to be associated with 9.9% (12/121; 95% CI, 4.6–15.2) and 27.6% (27/98; 95% CI, 18.7–36.4) cases, respectively. Of the 27 MDR isolates obtained from previously treated patients, 20 were found in treatment failure (20/39), five in relapse (5/21), and two in defaulter cases (2/13) (Table 3).

**Table 3:** First line anti-tuberculosis drug resistance stratified by previous treatment history

		**Previously treated cases**				
		**Total**	**Relapse**	**Treatment failure**	**Return after default**	**Unknown**
		**N**	**N**	**N**	**N**	**N**
**Total patients with DST**		98	21	39	13	25
**I**	**Any resistance to H**	46	9	22	4	11
**III**	H + R	6	2	4	0	0
	H + R + E	7	0	6	1	0
	H + R + S	5	0	4	1	0
	H + R + E + S	9	3	6	0	0
**Total MDR among previously treated cases**		**27**	**5**	**20**	**2**	**0**

### HIV co-infection

A total of 9.23% (194/2100) patients were found to be seropositive for HIV. The percentage positivity among males and females was 9.89% (146/1476) and 7.69% (48/624) respectively. Of the 219 *M. tuberculosis* isolates, 20.1% (44) and 79.9% (175) isolates were from HIV seropositive and seronegative patients, respectively. Among the 44 HIV seropositive patients there were 12 (27.3%), while in the 175 HIV seronegative patients there were 27 (15.4%) MDR isolates (Table 4). We applied multivariable regression analysis to see the association of MDR-TB with HIV status after controlling the effects of previous treatment status, age, and sex, and the association was found to be significant with odd’s ratio of 2.3 (95% CI, 1.000-5.350; p-value, 0.05) (Table 5). Of the remaining 32 (72.7%) strains from HIV positive patients, 18 (56.3%) isolates were susceptible to all the first line drugs, while among the 148 HIV seronegative patients which were not MDR, 80 (47.7%) isolates were susceptible to all the first line drugs.

**Table 4:** Anti-tuberculosis drug resistance stratified by patient HIV status

**DST results**		**HIV status**	
		**Positive (N = 44)**	**Negative (N = 175)**
**H + R**	Resistant to both	12	27
	Not resistant to both	32	148
**H**	Resistant	20	58
	Sensitive	24	117
**R**	Resistant	12	27
	Sensitive	32	148
**E**	Resistant	7	45
	Sensitive	37	130
**S**	Resistant	13	55
	Sensitive	31	120

**Table 5:** Multivariable regression model showing association of MDR-TB with HIV status after controlling the effects of age, sex, and previous treatment status

**Parameter estimates**								
**MDR(a)**		**B**	**Std. Error**	**Wald**	**df**	**p-value**	**Odd’s Ratio**	**95% CI for Odd’s Ratio**
**+ VE**	**Intercept**	−1.777	0.876	4.118	1	0.042	-	-
	**Sex**	0.284	0.415	0.467	1	0.494	1.328	0.589-2.997
	**Treatment**	1.380	0.397	12.066	1	0.001	3.976	1.825-8.664
	**Age**	−0.030	0.015	3.879	1	0.049	0.971	0.942-1.000
	**[C_HIV = 1]**	0.839	0.428	3.842	1	0.050	2.313	1.000-5.350
	**[C_HIV = 2]**	0(b)	-		0	-	-	-

a – The reference category is: - VE, b – This parameter is set to zero because it is redundant, CI – confidence interval, df – degree of freedom.

### Socio-demographic data, clinical presentation, and radiological picture of the TB cases

Among the 219 *M. tuberculosis* isolates, 155 (70.7%) and 64 (29.3%) were obtained from males and female patients, respectively. The mean (median, range) age of the patients was 36.6 years (35, 15–74), and 32.7 years (35, 4–75) for males and females respectively (p = 0.53). Of the 219 patients, 66.9% were from a lower socioeconomic background while 33.04% were of upper socioeconomic status, however, the prevalence of MDR-TB was found to be 19.5% and 23.7% in lower and upper socioeconomic groups, respectively, and the difference was not statistically significant (p = 0.3373). Among the patients, 91.6% presented with cough (mean duration, 4.8 months; range, 2 days to 48 months), 76.2% with fever (mean duration, 5.2 months; range, 7 days to 48 months), 73.2% with breathlessness, 68.8% with weight loss, 57.5% with chest pain, and 31.5% presented with hemoptysis. Radiology data was available for 49 patients, of which there was consolidation in 34 patients, cavity in 5, collapse in 3, pleural effusion in 3, fibrosis in 2, military shadows in 1, calcification in 1, and normal radiology picture in 4 patients.

## Discussion

The present study demonstrated a high prevalence of drug resistance amongst pulmonary TB isolates of *M. tuberculosis* from north India. In newly diagnosed cases the resistance to any of the first line drugs was observed to be 26.4% to isoniazid, 9.9% to rifampicin, 14.9% to ethambutol, and 28.1% to streptomycin. This is in agreement with reports from different parts of the country showing high resistance to isoniazid (32.9%), rifampicin (11.8%), and streptomycin (14.9%), but in contrast with a few other studies reporting low resistance to the tune of 3.2%, 0.5%, and 4.8% to isoniazid, rifampicin, and streptomycin respectively [5]. There was a high level of drug resistance in previously treated cases for any of the first line drugs i.e. 46.9% to isoniazid, 27.6% to rifampicin, 33.7% to ethambutol, and 34.7% to streptomycin. Other studies from India have also shown high resistance to isoniazid (47–87.1%) and rifampicin (12.6-80.6%) in previously treated cases; however, the number of isolates in most of the studies was small so the high resistance reported cannot be extrapolated to the whole country [5].

There were 39 cases of MDR-TB found in the study with an overall prevalence of 17.8% (39/219). In new cases a prevalence of 9.9% MDR-TB was observed which is higher than the WHO estimated prevalence of 2.1% cases of MDR-TB among the notified newly diagnosed pulmonary TB cases in India in 2010. However, the reports of prevalence of MDR-TB in newly diagnosed cases vary from 0.6 to 24% from different regions of the country [11-13]. Similarly, among the previously treated group multidrug resistance was seen to be 27.6%, which is higher than the WHO estimate of 15% MDR-TB in notified retreatment cases in India in 2010, though other studies from different regions of the country report MDR-TB varying from 11.8 to 47.1% [14-17]. The data of MDR-TB from other regions of the world ranges from 2.1%, 4.9%, and 12%, in newly diagnosed cases to 12%, 23%, and 37% in re-treatment cases in the Region of the Americas, Western Pacific Region, and European Region respectively [1]. The wide range of resistance data from different parts of India indicates the lack of uniform surveillance methodologies across the country. Also, the sample size in most of the studies is small and do not reflect a wholesome picture of the resistance data. On the other hand, this difference in the resistance patterns may be true to some extent, considering the wide geographical and administrative divisions of the country reflecting the gaps in implementation of the national TB control programs; although this needs to be studied in large multi-centric surveillance studies using uniform methodologies to obtain the drug resistance data. The high prevalence in our study could be attributed to the fact that it was carried out at a tertiary care hospital where patients are being referred from different parts of north India. Surprisingly, we did not find any isolate which was XDR-TB though XDR-TB has been reported from India and other parts of the world [18-22].

Of the total 2100 cases, there was a seropositivity of 9.23% for HIV, while among the 219 patients with TB there were 20.1% seropositive cases. In our study, a higher prevalence of HIV in TB patients was found as compared to the WHO estimate of a prevalence of 9% of HIV in TB patients in India for 2010, which may possibly be due to clustering of referred cases at our tertiary care hospital. However, similar to our findings a few studies from other parts of the country have shown higher seroprevalence of HIV in TB approaching to 17% and 20.39% from Chennai and Pune respectively [23,24]. There was a significantly higher number of MDR *M. tuberculosis* isolates in the HIV seropositive group i.e. 27.3%, as compared to 15.4% in HIV seronegative group with an odd’s ratio of 2.3 (95% CI, 1.000-5.350; p value, 0.05).

In the present study, a majority of the patients with TB were from a lower socioeconomic background (66.9%), however the prevalence of MDR-TB in these patients was found to be 19.5% which was not significantly different from the prevalence of 23.7% among the patients of upper socioeconomic status. This indicates that although TB is more common in poor patients, yet poverty was not a determining factor for the occurrence of MDR-TB, although the sample size was not sufficient to give this observation a statistical significance. In a recent study conducted in South India, Gupta et al. observed a significantly higher prevalence of pulmonary TB in labourers, followed by white-collar workers, retired and unemployed, household workers and students, however, they have not looked into drug resistance patterns in these patients [25]. The increased incidence of pulmonary TB among socio-economically lower classes can be attributed to lower education level and poverty [26,27], although the true prevalence of TB in poor sections of the society may be higher than estimated because such patients have limited access to hospital medical services and a high mortality rate [25].

## Conclusion

In conclusion, in this study we demonstrate a high prevalence of drug resistance amongst pulmonary TB isolates of *M. tuberculosis* from northern India. There was a high prevalence of MDR-TB in both new and previously treated patients as compared to the WHO estimated prevalence of MDR-TB in India in 2010, however, we did not find any XDR-TB isolate. We also observed a high prevalence of HIV among the TB patients and significant association of MDR-TB with HIV seropositive patients as compared to seronegative patients. Although TB was found to be more common in patients with poor socioeconomic background, yet there was no significant difference in the distribution of MDR-TB in different social strata.

The data from the present study should be extrapolated with certain limitations given the diverse population from which these estimates were made. The enrolled patients presented to our tertiary care centre from several states of north India, and many of our patients are partially treated previously, before being referred to our centre to seek additional care. This may have resulted in higher proportions of patients with MDR-TB at the time they were initially diagnosed at our centre, as patients with less resistant disease may not have needed to seek additional care. The higher prevalence of HIV in our TB patients compared to the WHO estimates for India may again possibly be due to clustering of referred cases at our tertiary care centre.

Overall, these findings emphasize the importance of continuing the systematic surveillance of *M. tuberculosis* isolates to monitor the trends of drug resistance in different patient categories as well as its association with HIV across the country to timely modify and strengthen the national programs in order to prevent the emergence of MDR-TB strains and avert the threat of XDR-TB.

## Competing interests

All authors declared no conflict of interest.

## Authors’ contributions

Conceived and designed the study: SS, MS, DG, AW; Performed the study: SKD, RY, HS, KS. Analyzed the data: SS, AM, SKD. Contributed reagents/materials/analysis tools: SS, MS. Enrolled patients: DG, AW. Wrote the paper: SS, AM, SKD. Critical review of the paper: All. All authors read and approved the final manuscript.

## Pre-publication history

The pre-publication history for this paper can be accessed here:

http://www.biomedcentral.com/1471-2334/13/137/prepub
